# MMP‐13 deletion decreases profibrogenic molecules and attenuates *N*‐nitrosodimethylamine‐induced liver injury and fibrosis in mice

**DOI:** 10.1111/jcmm.13304

**Published:** 2017-08-07

**Authors:** Joseph George, Mikihiro Tsutsumi, Mutsumi Tsuchishima

**Affiliations:** ^1^ Department of Medicine Division of Molecular Medicine College of Physicians and Surgeons Columbia University New York NY USA; ^2^ Department of Hepatology Kanazawa Medical University Uchinada Ishikawa Japan

**Keywords:** connective tissue growth factor, *N*‐nitrosodimethylamine, NDMA, MMP‐13, hepatic fibrosis

## Abstract

Connective tissue growth factor (CTGF) is involved in inflammation, pathogenesis and progression of liver fibrosis. Matrix metalloproteinase‐13 (MMP‐13) cleaves CTGF and releases several fragments, which are more potent than the parent molecule to induce fibrosis. The current study was aimed to elucidate the significance of MMP‐13 and CTGF and their downstream effects in liver injury and fibrosis. Hepatic fibrosis was induced using intraperitoneal injections of *N*‐nitrosodimethylamine (NDMA) in doses of 10 μg/g body weight on three consecutive days of each week over a period of 4 weeks in both wild‐type (WT) and MMP‐13 knockout mice. Administration of NDMA resulted in marked elevation of AST, ALT, TGF‐β1 and hyaluronic acid in the serum and activation of stellate cells, massive necrosis, deposition of collagen fibres and increase in total collagen in the liver of WT mice with a significant decrease in MMP‐13 knockout mice. Protein and mRNA levels of CTGF, TGF‐β1, α‐SMA and type I collagen and the levels of MMP‐2, MMP‐9 and cleaved products of CTGF were markedly increased in NDMA‐treated WT mice compared to the MMP‐13 knockout mice. Blocking of MMP‐13 with CL‐82198 in hepatic stellate cell cultures resulted in marked decrease of the staining intensity of CTGF as well as protein levels of full‐length CTGF and its C‐terminal fragments and active TGF‐β1. The data demonstrate that MMP‐13 and CTGF play a crucial role in modulation of fibrogenic mediators and promote hepatic fibrogenesis. Furthermore, the study suggests that blocking of MMP‐13 and CTGF has potential therapeutic implications to arrest liver fibrosis.

## Introduction

Hepatic fibrosis is a result of an abnormal wound healing in response to chronic liver injury from various causes 1, 2. The pathogenesis of hepatic fibrosis is a dynamic and complex process that involves various cell types in the hepatic tissue including the hepatic progenitor cells 3, 4. The chronic hepatocyte injury leads to the activation of resting hepatic stellate cells (HSCs) into myofibroblast‐like cells with the characteristic expression of smooth muscle actin filaments 5, 6. The activated hepatic stellate cells initiate a series of signalling and transcriptional events that triggers the pathogenesis of hepatic fibrosis 7, 8.

*N‐*Nitrosodimethylamine (NDMA), also known as dimethylnitrosamine (DMN), is a by‐product of several industrial processes and a suspected human carcinogen and highly toxic to the liver. It was demonstrated that NDMA‐induced model of hepatic fibrosis in rats is an appropriate and suitable animal model for studying biochemical and molecular changes that associated with the pathogenesis of hepatic fibrosis and cirrhosis of human beings 9, 10. This model also depicts several decompensating features of alcoholic fibrosis and cirrhosis such as portal hypertension, ascites, hypoproteinemia and biochemical alterations 11, 12. Recently, NDMA‐induced mouse model of liver injury has been used to study the interaction between pro‐fibrotic and anti‐fibrotic molecules during the development of hepatic fibrosis 13. The extensive oxidative stress and production of reactive oxygen species (ROS) during detoxification of NDMA triggers acute liver injury and subsequent fibrosis.

Matrix metalloproteinases (MMPs) are a gene family of zinc‐ and calcium‐dependent endopeptidases that are capable of degrading extracellular matrix (ECM) components including all forms of native collagens, and play a key role in remodelling the ECM in both physiological and pathological conditions 14, 15. The major interstitial collagenases that degrade native fibrillar collagens are MMP‐1 and MMP‐13. However, mice and rats do not possess a homologue to human MMP‐1. Thus, MMP‐13 is responsible for the metabolic turnover of major fibrillar collagens in mice 16, and transgenic expression of MMP‐1 inhibited myocardial fibrosis and prevented heart failure in a mouse model 17.

Connective tissue growth factor (CTGF), a 38‐kD cysteine‐rich protein, is a multifunctional protein involved cell proliferation and tissue remodelling, and induces by transforming growth factor‐β1 (TGF‐β1) 18, 19, 20. CTGF plays a central role in the pathogenesis of hepatic fibrosis by triggering activation and transformation of quiescent hepatic stellate cells into myofibroblasts, and it also stimulates the production of CTGF itself 21, 22. Activated stellate cells over‐produce collagens, fibronectin and laminin under stimulation with CTGF 23. The two major MMPs, that is MMP‐1 and MMP‐13, cleave full‐length CTGF into the N‐terminal and C‐terminal fragments of similar molecular weights 19, 24. Due to the absence of MMP‐1 in adult mice 25, 26, MMP‐13 is primarily responsible for the cleavage of full‐length CTGF into its fragments that are more potent to induce hepatic fibrogenesis, as it has been reported that the N‐terminal fragment of CTGF mediates myofibroblast differentiation and collagen synthesis and the C‐terminal fragment stimulates fibroblast proliferation 27. The N‐terminal fragment also acts as downstream mediator of TGF‐β1 and increases in fibrotic processes 27, 28. Uchinami *et al*. 29 reported that MMP‐13 contributes to accelerating fibrogenesis by mediating the initial inflammation of the liver. However, the mechanism by which MMP‐13 promotes fibrogenesis through mediating the initial inflammation of the liver is not clear. In the current study, we attempted to elucidate some of the mechanisms behind this observation with a specific emphasis to CTGF and its cleaved subunits. NDMA was used to induce the liver injury in wild‐type (WT) and MMP‐13 knockout (KO) mice for the purpose. Cultured rat hepatic stellate cells were used for *in vitro* studies.

## Materials and methods

### MP‐13 knockout and wild‐type mice

The MMP‐13 knockout (KO) mice on a 129/Sv genetic background were generated by microinjection of embryonic stem cells into C57BL/6J blastocytes as described before 30, 31. Adult MMP‐13 KO mice were back‐crossed with C57BL/6J strain for at least five times. MMP‐13 KO and wild‐type (WT) littermates were generated from the intercross between MMP13^+/−^ mice, and both male and female mice were used for the experiments. Genotyping was performed by PCR analysis using DNA obtained from tail biopsies of two‐week‐old pups. The KO mice exhibited a normal lifespan with sufficient fertility and did not exhibit any gross abnormalities after maturation, except growth retardation due to defects in the growth plate during development 30, 31. Histopathological examination of paraffin sections stained with haematoxylin and eosin and Masson's trichrome for collagen from the liver, lung, heart, kidney and uterus showed no pathological alterations or collagen deposition in adult MMP‐13 KO mice.

The animal experiments were carried out with the *Guide for the Care and Use of Laboratory Animals* prepared by the National Academy of Sciences and published by the US National Institutes of Health (NIH Publication No. 86‐23, revised 1996) and also in compliance with the Institutional Animal Care and Use Committee (IACUC). Both male and female animals were used for the induction of liver injury. All animals received humane care according to the criteria outlined in the manual.

### Induction of liver injury and fibrosis in WT and MMP‐13 KO mice

Both WT and MMP‐13 KO mice around three months of age, weighing approximately 25 g, were used for the experiment. The animals were divided into four groups of eight animals each. Hepatic fibrosis was induced by serial intraperitoneal (i.p.) injections of *N*‐nitrosodimethylamine (NDMA) in doses of 10 μg/g body weight on three consecutive days of every week over a period of 4 weeks in one group of both WT and MMP‐13 KO mice. NDMA was procured from Sigma‐Aldrich (St. Louis, MO, USA) and diluted appropriately with 0.15 mol/l sterile NaCl. Control groups received similar injections without NDMA. The injections were given without anaesthesia. All the animals were provided with mice feed and water available *ad libitum*. A few animals died in both WT and MMP‐13 KO NDMA‐treated groups due to massive hepatic necrosis. However, the experiment was repeated with additional animals and the total number of animals was eight in each group at the end of the study. The animals were killed on day 28 from the beginning of NDMA administration along with control animals after anesthetization with a mixture of ketamine and xylazine. Blood and liver tissue were collected and processed for biochemical and molecular biological studies.

### Evaluation of NDMA‐induced liver injury and hepatic fibrosis

NDMA‐induced hepatic injury and fibrosis were assessed by observing histology of the liver stained with haematoxylin and eosin and Masson's trichrome. The median lobe of the liver tissue was cut into approximately 3‐mm slices and fixed in 10% phosphate‐buffered formalin. The fixed liver tissues were processed in an automatic tissue processor optimized for liver, embedded in paraffin blocks and cut into sections of 5 μm thickness. haematoxylin and eosin and Masson's trichrome staining were carried out using standard methodology (#K037; Poly Scientific, Bay Shore, NY, USA). The staining intensity was examined using an Olympus microscope (Olympus Corporation, Tokyo, Japan) and photographed. The number of activated stellate cells was evaluated using immunohistochemical staining of α‐smooth muscle actin (α‐SMA), which is a marker for initiation of hepatic fibrosis.

### Determination of hydroxyproline and total collagen content in the liver

Hydroxyproline and total collagen content in the liver tissue was determined as a biochemical parameter to assess the degree of hepatic fibrosis after the treatment with NDMA. Exactly, 50‐mg wet liver tissue was hydrolysed in 6 N HCl in sealed tubes at 110°C for 16 hrs. The hydrolysed samples were evaporated to dryness in a boiling water bath to remove acid, and the residue was dissolved in 5 ml of distilled water. It was then treated with activated charcoal, vortexed well and filtered through Whatman No. 1 filter paper. Hydroxyproline content in the clear filtrate was measured as described before 32. In brief, 1 ml of sample containing 1–5 μg hydroxyproline was mixed with 1 ml of freshly prepared chloramine‐T solution and allowed to stand for 20 min. It was then mixed with 1 ml of 3.15 M perchloric acid and left for 5 min. Next, 1 ml of freshly prepared *p*‐dimethylaminobenzaldehyde was added, vortexed well and incubated in a water bath at 60°C for 20 min. Absorbance of the colour development was measured in a spectrophotometer at 560 nm. The concentration of hydroxyproline in the samples was determined using a known standard of *L*‐hydroxyproline (Sigma‐Aldrich) solution. The total collagen content in the liver tissue was calculated by multiplying the hydroxyproline content by the factor 7.46 as described previously 2.

### Measurement of ALT and AST

Blood was allowed to clot by standing for 3–5 hrs and serum separated by the conventional method. Serum alanine transaminase (ALT) and aspartate transaminase (AST) were measured using an auto‐analyser and presented as International Units per Liter.

### Immunohistochemical staining for α‐SMA and CTGF

The immunohistochemical staining for α‐SMA and CTGF was carried out on paraffin‐embedded tissue using a universal staining kit (#85‐9943; Invitrogen, Carlsbad, CA, USA). The paraffin liver sections were deparaffinized and hydrated with water. The clear sections were treated with primary antibodies against α‐SMA (#18‐0106; Invitrogen) or CTGF (#ab6992; Abcam, Cambridge, MA, USA) and non‐immune control IgG in a moisturized chamber at 4°C overnight. They were washed three times in cold phosphate‐buffered saline (PBS) and incubated with biotinylated anti‐rabbit or antimouse secondary antibodies for 1 hr at room temperature. The slides were washed again and treated with horseradish peroxidase‐labelled streptavidin and incubated for another 1 hr. Colour was developed using 3% 3‐amino‐9‐ethylcarbazole (AEC). The stained sections were counterstained Mayer's haematoxylin and examined under a Nikon microscope attached with a Spot RT Slider digital camera (Meyer Instruments, Houston, TX, USA) and photographed. The images were quantified using Image‐pro discovery software (Media Cybernetics, Silver Spring, MD, USA).

### Measurement of IL‐6, hyaluronic acid and TGF‐β1 in serum

Interleukin‐6 (IL‐6) in mouse serum was measured using sandwich ELISA kit (#ab100712; Abcam) following manufacturer's protocol. In brief, 100 μl serum or recombinant IL‐6 standard was added to a 96‐well plate coated with mouse IL‐6 antibody and incubated for 2.5 hrs at room temperature with gentle shaking. After several washing, biotinylated IL‐6 second antibody was added followed by HRP‐conjugated streptavidin and TMB (tetramethylbenzidine) substrate. The intensity of the coloured product was measured on a microplate plate reader at 450 nm. Hyaluronic acid (HA) levels in mouse serum were determined using an ELISA‐based sandwich HA‐binding protein assay kit (Chugai Diagnostics Science, Tokyo, Japan) as per the manufacturer's instructions, which follow the method of Chichibu *et al*. 33. TGF‐β1 levels in mouse serum were determined using a sandwich enzyme assay kit (R&D Systems, Minneapolis, MN, USA) according to the manufacturer's instructions. In brief, 50 μl of 60‐fold diluted serum samples was added to a microplate pre‐coated with TGF‐β1 monoclonal antibody followed by 50 μl of assay diluent provided in the kit. The samples were mixed gently and incubated for 2 hrs at room temperature. The wells were washed 3–5 times with wash buffer included in the kit, and 100 μl of TGF‐β1 secondary antibody conjugate was added, and incubated for 2 hrs. Finally, 100 μl of TMB substrate solution was added and incubated for 30 min. in the dark for colour development. The reaction was arrested with stop solution, and the intensity of the colour was determined on a microplate reader at 450 nm.

### Gelatin zymography for MMP‐9 and MMP‐2

The activity of MMP‐9 and MMP‐2 in the liver tissue from WT and MMP‐13 KO mice was analysed using gelatin zymography 34. About 100 mg of fresh liver tissue was homogenized in 1 ml of 50 mM Tris‐HCl buffer (pH 8.0) containing 150 mM NaCl and 1% Triton X‐100. The homogenates were centrifuged at 5000 × *g* for 30 min. at 4°C, and the supernatants were collected. After equalizing the protein concentration, 30 μg protein was loaded to a 10% polyacrylamide gel containing 0.1% bovine skin gelatin (#G8150; Sigma‐Aldrich) under non‐reducing conditions. The gels were washed twice (30 min./wash) in 2.5% Triton X‐100 at room temperature and then incubated overnight at 37°C in 50 mM Tris‐HCl buffer (pH 7.6) containing 10 mM CaCl_2_. The gels were stained with Coomassie brilliant blue R‐250 for 30 min. and destained until clear transparent bands were developed and photographed. The bands were quantified using Gel‐Pro analyzer software (Media Cybernetics).

### Western blotting for MMP‐9 and MMP‐2

Proteins (20 μg/lane) prepared from the liver tissue were resolved on a 7.5% SDS‐PAGE under reducing conditions. The proteins were electrotransferred onto an activated polyvinylidene fluoride (PVDF) membrane (Millipore, Bedford, MA, USA). The membranes were incubated with 5% skim milk to block non‐specific reactions and reacted overnight on a rocker at 4°C with antibodies against MMP‐9 (#19016; Millipore) or MMP‐2 (#4022; Cell Signaling Technology, Danvers, MA, USA) that recognize the mouse antigens. After washing in PBS, they were further incubated with horseradish peroxidase (HRP)‐conjugated secondary antibody (Biomeda, Foster City, CA, USA) at room temperature for 2 hrs. The membranes were then treated with chemiluminescence reagent (#RPN2232; ECL Prime, GE Healthcare, Piscathaway, NJ, USA) exposed to BioMax XAR autoradiography film (Kodak, New Haven, CT, USA). The membranes were reprobed for β‐actin to demonstrate equal loading of protein samples in each lane.

### Quantitative real‐time PCR for MMP‐9

Control and NDMA‐treated WT and MMP‐13 KO mouse liver tissues were snap‐frozen in liquid nitrogen and stored at −80°C. The total RNA was isolated using RNeasy kit (Qiagen, Valencia, CA, USA). The purity of the isolated RNA was evaluated on a spectrophotometer and with agarose gel electrophoresis. Mouse MMP‐9 primer set was designed using Primer 3 software (NHGRI, NIH, USA), and the primer sequences for mouse MMP‐9 gene (NM_013599) were as follows: forward 5′‐CGT CGT GAT CCC CAC TTA CT‐3′ and reverse 5′‐AAC ACA CAG GGT TTG CCT TC‐3′. Quantitative PCR was performed using a one‐step RT‐PCR kit with SYBR green (Bio‐Rad, Hercules, CA, USA) on a real‐time PCR machine (iCycler iQ5; Bio‐Rad). The reaction conditions were set as: cDNA synthesis, 10 min. at 50°C; reverse transcriptase inactivation at 95°C for 5 min.; PCR cycling and detection at 95°C for 10 sec.; and data collection at 56°C for 30 sec. About 100 ng of isolated RNA was used in the experiment.

### Real‐time PCR and semiquantitative RT‐PCR for the major molecules involved in hepatic fibrosis

Total RNA was isolated from the flash‐frozen liver tissue employing PureLink RNA Mini Kit (Invitrogen) in combination with Trizol as per manufacturer's instructions. The gene specific primers for CTGF, TGF‐β1, α‐SMA, type I collagen (α1 chain) and Glyceraldehyde 3‐phosphate dehydrogenase (GAPDH) were designed using Beacon Designer software (Premier Biosoft International, Palo Alto, CA, USA). Details of the primer sets used are provided in supplementary Table 1. The same primer sets were used for both real‐time PCR and semiquantitative RT‐PCR. The cDNA was synthesized with 1–2 μg of isolated total RNA using Sprint RT 8‐well strips (Clontech, Mountain View, CA, USA) in a total volume of 20 μl in RNAse‐free H_2_O at 42°C for 60 min. Real‐time quantitative PCR (qPCR) was carried out with FastStart SYBR Green Master (Roche Applied Science, Indianapolis, IN, USA) on ABI 7500 Real‐Time PCR System (Applied Biosystems, Foster City, CA, USA). The synthesized cDNA was diluted to 20‐fold with RNAse‐DNAse‐free water. The qPCR reaction was carried out with 5 μl of diluted cDNA and 5 μl of SYBR Green master mix containing gene specific primers in a 384‐well plate (total volume 10 μl). All samples were run in triplicates. The qPCR parameters were set as follows; denature 1 cycle, amplification 45 cycles, melting curve 1 cycle and cooling 1 cycle. All data were normalized to GAPDH gene.

**Table 1:** Sequences of the primers used in semiquantitative and real‐time RT‐PCR

Transcript	GenBank number	Primer sequence	Position	Length (mer)	Product size (bp)
CTGF	BC006783	5′–CAAAGCAGCTGCAAATACCA–3′ 5′–GGCCAAATGTGTCTTCCAGT–3′	551F 770R	20 20	220
TGF‐β1	BC013738	5′–TGAGTGGCTGTCTTTTGACG–3′ 5′–tCTCTGTGGAGCTGAAGCAA–3′	893F 1185R	20 20	293
α‐SMA	X13297	5′–CTGACAGAGGCACCACTGAA–3′ 5′–CATCTCCAGAGTCCAGCACA–3′	368F 527R	20 20	160
Collagen type I	BC059281	5′–AAGAGGCGAGAGAGGTTTCC–3′ 5′–AGAACCATCAGCACCTTTGG–3′	2101F 2344R	20 20	244
GAPDH	BC145810	5′–AACTTTGGCATTGTGGAAGG–3′ 5′–ACACATTGGGGGTAGGAACA–3′	536F 758R	20 20	223

F: forward primer; R: reverse primer.

Semiquantitative RT‐PCR was performed for all of the above molecules for additional evaluation of their mRNA expression. All of the above primer sets were transcribed with 100 ng of isolated total RNA using SuperScript one‐step RT‐PCR system with Platinum Taq DNA polymerase (Invitrogen) on a thermocycler (GeneAMP PCR Systems 9700; Applied Biosystems). The reaction conditions were set as: cDNA synthesis at 50°C for 30 min.; inactivation at 94°C for 2 min.; PCR amplification of 35 cycles; denaturation at 94°C for 20 sec.; annealing at 56°C for 30 sec.; chain extension at 72°C for 45 sec.; and a final chain extension at 72°C for 10 min. The PCR products were resolved on 1.5% agarose gel containing ethidium bromide and examined with a transilluminating UV system (Alpha Innotech Corporation, San Leandro, CA, USA) connected with a computer. The PCR product images were quantified employing Gel‐Pro analyzer software (Media Cybernetics).

### Western blotting for the major molecules involved in pathogenesis of hepatic fibrosis

To examine the expression of the molecules involved in the pathogenesis of hepatic fibrosis in WT and MMP‐13 KO mice after treatment with NDMA, 100 mg of liver tissue was homogenized in 1 ml of cold 50 mM Tris‐HCl buffer (pH 8.0) containing 150 mM NaCl, 1 mM Ethylenediaminetetraacetic acid (EDTA) and 1% Triton X‐100. The protease inhibitors, 0.5 mM phenylmethylsulfonyl fluoride, 5 μg/ml aprotinin and 1 μg/ml pepstatin were added to the buffer just prior to use. The homogenates were centrifuged at 10,000 × *g* for 10 min. at 4°C, and the supernatant was collected. Protein concentrations in the supernatants were determined using a protein assay kit (#23227; Pierce Biotechnology, Rockford, IL, USA), and the samples were stored at −20°C until used. The proteins were denatured and resolved on 4–20% SDS‐polyacrylamide gradient gel (Bio‐Rad) and electrotransferred to an activated PVDF membrane. Non‐specific binding was blocked with 5% non‐fat dry milk, and the membranes were incubated overnight on a rocker at 4°C with specific antibodies. Antibody to tissue inhibitor of metalloproteinase (TIMP)‐1 (#sc‐5538) was procured from Santa Cruz Biotechnology (Santa Cruz, CA, USA). The antibodies to cleaved TGF‐β1 (#ab9758) and α‐SMA (#ab5694) were from Abcam, and the antibody to type I collagen was from Millipore (#AB765P). The polyclonal antibodies that detect the full‐length CTGF and its C‐terminal fragments were raised in rabbits by injecting bovine serum albumin (BSA)‐conjugated oligopeptide corresponding to the amino acid sequence of the CTGF C‐terminal domain (I^257^RTPKISKPIKFELSG^272^C) and previously characterized (24). IgG was isolated from the antisera by DEAE‐Sephacel column chromatography and purified. After incubation with the primary antibodies, the membranes were washed thrice and treated with respective HRP‐conjugated secondary antibodies (Biomeda) at room temperature for 2 hrs. They were washed again, treated with enhanced chemiluminescence reagent (GE Healthcare, Piscataway, NJ, USA), exposed to Kodak autoradiography film (BioMax XAR, New Haven, CT, USA), and developed. The membranes were reprobed using Western reprobe buffer (#786‐119; Gbiosciences, St. Louis, MO, USA) for GAPDH content with anti‐GAPDH monoclonal antibody (#NB300‐221; Novus Biologicals, Littleton, CO, USA) to show that equal amounts of protein samples were loaded in all lanes. The Western blotting images were quantified using Gel‐Pro analyzer software (Media Cybernetics).

### Inhibition of MMP‐13 in hepatic stellate cells

To examine whether inhibition of MMP‐13 could decrease active CTGF and TGF‐β1 protein levels *in vitro*, liver stellate cell cultures were treated with CL‐82198 (*N*‐[4‐(4‐Morpholinyl) butyl]‐2‐benzofurancarboxamide hydrochloride) (#ab141576; Abcam), which is a selective inhibitor of MMP‐13 35. Stellate cells were isolated from about one year old albino rats and cultured as reported before 21. The cells were cultured in 50:50 mixture of DMEM and Ham's F12 medium (Invitrogen) containing 10% foetal bovine serum (FBS; Invitrogen) and antibiotics (100 units of penicillin G and 100 μg of streptomycin/ml of culture media) in a humidified incubator containing 5% CO_2_ on air at 37°C. About 80% confluent cells were harvested using Trypsin‐EDTA (Invitrogen) and sub‐cultured into 4‐well glass microscopic chamber slides (CAT# 154526PK, Lab‐Tek, Thermo Fisher Scientific, Waltham, MA, USA) treated with 0.1% collagen solution. Another set was cultured into 100‐mm culture dishes for Western blotting analysis. After 48 hrs, the media were replaced with reduced serum medium (2% FBS) containing 10 μM CL‐82198 (final concentration) (reported IC_50_ = 3.2 μM) 36 and incubated for another 48 hrs. A second set of cultures were prepared in the same way without CL‐82198 and served as control. The medium was discarded, washed the slides twice with PBS, and the cells were fixed in 1:1 methanol and ethanol mixture at −20°C for 10 min. The cells were washed with PBS and staining was performed for CTGF. The staining intensity was quantified using Image‐Pro Plus software (Media Cybernetics) and represented as square microns.

The treated cells in culture dishes were washed well with cold PBS and harvested with 1 ml of radio‐immunoprecipitation assay (RIPA) buffer prepared freshly with protease inhibitors. The cells were centrifuged at 12,000 *× g* for 10 min. at 4°C in a micro‐centrifuge (Sorvall; Thermo Fisher). The cell pellet was collected and suspended in RIPA buffer containing protease inhibitors, depends on the size of the pellet and sonicated gently in an Omni Ruptor 400 Ultrasonic cell disruptor (Omni International, Kennesaw, GA, USA). The cell suspension was centrifuged at 14,000 *× g* for 10 min. at 4°C, and the supernatants were collected. Protein concentration in the supernatant was determined and Western blotting for full‐length CTGF and its C‐terminal fragments and cleaved TGF‐β1 was carried out as described above. Finally, the Western blot membranes were treated with enhanced chemiluminescence reagent (#RPN2232; ECL Prime, GE Healthcare, Piscataway, NJ, USA) and the resultant chemiluminescence was detected using Fuji ImageQuant LAS 4000 (GE Healthcare) and the digitized images were saved on a computer. The membranes were reprobed using Western reprobe buffer (Gbiosciences), and Western blotting was performed for β‐actin with antibody (#A5441; Sigma‐Aldrich) to demonstrate that equal amount of protein had been loaded in each lane. The Western blotting images were quantified using Gel‐Pro analyzer software (Media Cybernetics).

### Statistical analysis

Arithmetic mean and standard deviation (S.D.) were calculated for all quantitative data. The data were statistically evaluated using paired Student's *t‐*test, and a value of *P* < 0.05 was considered as significant. Pearson's correlation coefficient analysis was used to evaluate the correlation between qPCR and semiquantitative PCR for the expression of major molecules involved in the pathogenesis of hepatic fibrosis.

## Results

### NDMA‐induced liver injury and fibrosis

**Figure 1:**
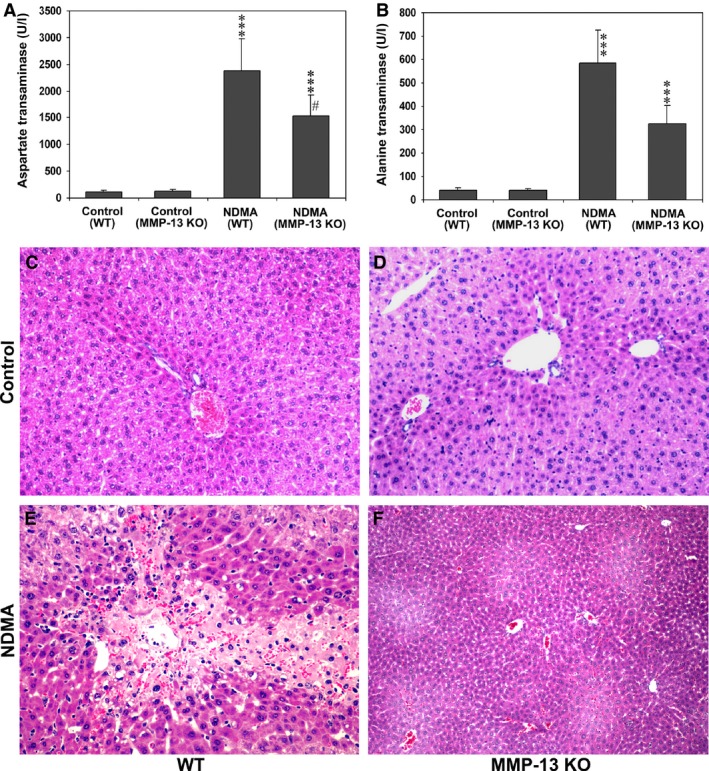
Serological and histological findings of NDMA‐induced liver injury in WT and MMP‐13 KO mice. (**A** and **B**) Serum levels of aspartate transaminase (AST) and alanine transaminase (ALT) in WT and MMP‐13 KO mice treated with or without NDMA. The data are mean ± S.D. of eight animals. ***P < 0.001 NDMA‐treated mice *versus* untreated control mice; ^#^ *P* < 0.001 NDMA‐treated WT *versus* MMP‐13 KO mice. (**C**–**F**) Haematoxylin and eosin staining of liver tissues from WT and MMP‐13 KO mice without NDMA treatment (**C** and **D**) and with NDMA treatment (**E** and **F**). Note that the marked contrast of hepatic necrosis with haemorrhage and inflammatory cell infiltration in NDMA‐treated WT and MMP‐13 KO mouse livers shown in E and F, respectively. Original magnification, ×100 for C, D, and E; ×40 for F.

Serum levels of AST and ALT were remarkably increased in WT and MMP‐13 KO mice that were administered with NDMA, whereas both control mouse groups untreated with NDMA showed only the baseline levels (Fig. 1A and B). When the mean serum levels were compared between NDMA‐treated WT and MMP‐13 KO mice, AST and ALT were significantly higher in WT mouse group than in MMP‐13 KO mouse group (*P* < 0.001) (Fig. 1A and B). Histologically, control WT mice without NDMA treatment showed normal lobular architecture with central veins and radiating hepatic cords (Fig. 1C), and there was no alteration in the liver tissue of control MMP‐13 KO mice (Fig. 1D). In contrast, administrations of NDMA to WT mice exhibited extensive hepatic necrosis with haemorrhage and infiltration of mononuclear cells and neutrophils (Fig. 1E). However, NDMA‐treated MMP‐13 KO mice showed only mild focal haemorrhagic necrosis of hepatocytes (Fig. 1F).

### Decrease of collagen content in MMP‐13 KO mice liver

**Figure 2:**
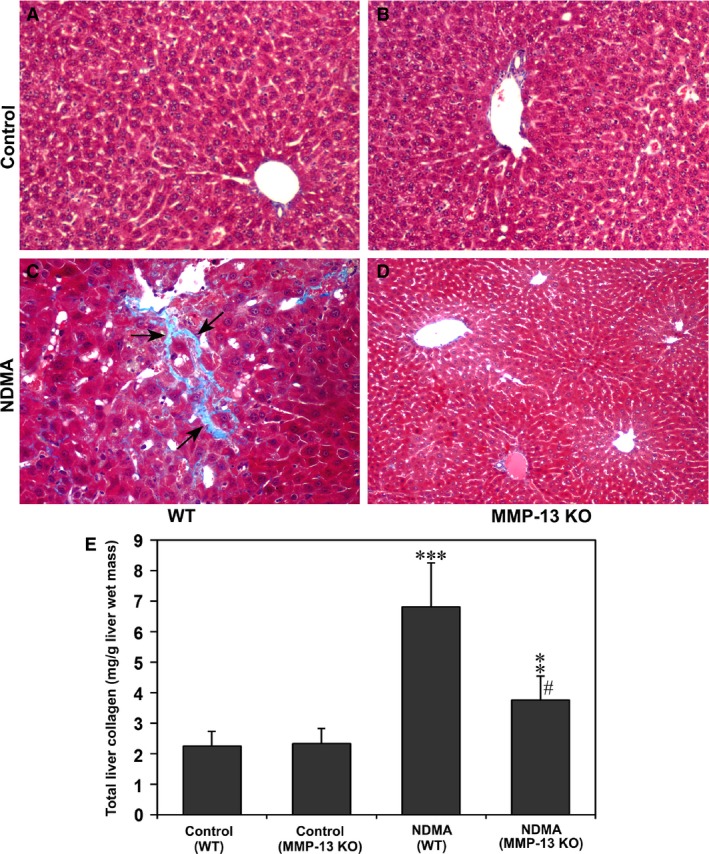
Masson's trichrome staining of liver tissues from WT and MMP‐13 KO mice without (**A** and **B**) and with NDMA treatment (**C** and **D**). Note the definite deposition of collagen fibres in NDMA‐treated WT mouse liver (**C**, arrows) and negligible staining in MMP‐13 KO mouse liver (**D**). Original magnification, ×100 for A, B, and C; ×40 for D. (**E**). Total collagen content in WT and MMP‐13 KO mice liver without and with NDMA treatment. The total content in the liver tissue was determined by estimating the hydroxyproline content, a characteristic imino acid present in collagen, and multiplied the value with 7.46. The data are mean ± S.D. of eight animals. ***P < 0.001 NDMA‐treated WT mice *versus* untreated WT mice and **P < 0.01 NDMA‐treated MMP‐13 KO mice *versus* untreated MMP‐13 KO mice; ^#^ *P* < 0.001 NDMA‐treated WT *versus* MMP‐13 KO mice.

Masson's trichrome staining did not show any collagen deposition in the liver parenchyma of either WT or MMP‐13 KO control mice (Fig. 2A and B). However, NDMA‐administered WT mice demonstrated deposition of thick collagen fibres (arrows) in the areas of bridging fibrosis (Fig. 2C), whereas such collagen deposition was not present in NDMA‐treated MMP‐13 KO mice (Fig. 2D).

The total collagen content in the liver tissue determined by estimating hydroxyproline, a characteristic imino acid present in collagen, was significantly increased (*P* < 0.001) in WT mice after the administration of NDMA (Fig. 2E). The increase was around 3‐fold compared to the total collagen content in WT control mice liver. The total collagen content in NDMA‐treated MMP‐13 KO mice was also significantly higher (*P* < 0.01) compared to non‐treated MMP‐13 KO mice (Fig. 2E). However, compared to the increased collagen level in NDMA‐treated WT mice liver, the collagen content in NDMA‐treated MMP‐13 KO mice was significantly lower (*P* < 0.001). There was no difference in the collagen content between WT and MMP‐KO control mice livers (Fig. 2E).

### Activation of hepatic stellate cells is attenuated in MMP‐13 KO mice

**Figure 3:**
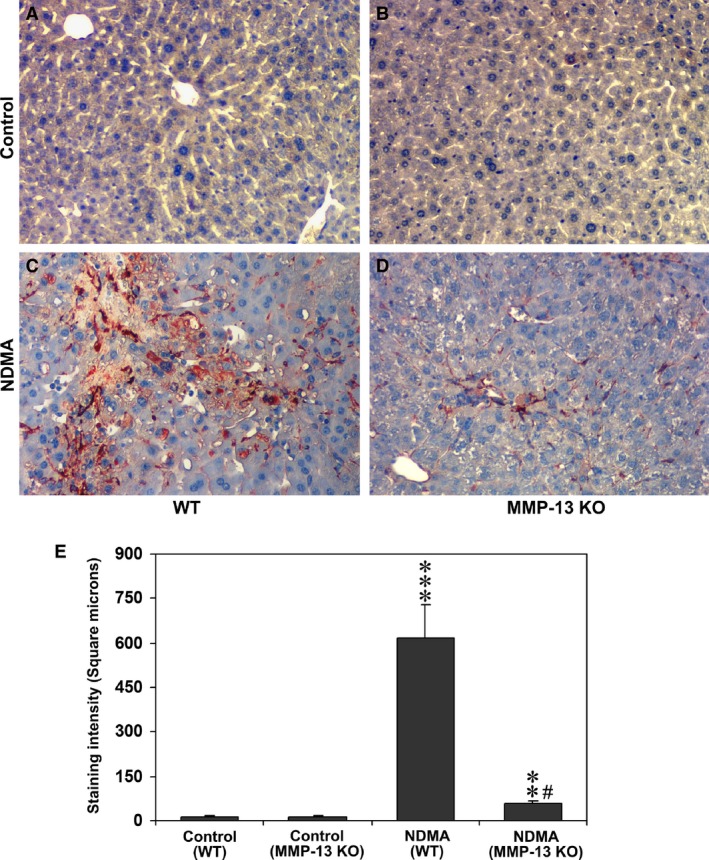
Immunohistochemical staining of α‐SMA in the liver tissues from WT and MMP‐13 KO mice without (**A** and **B**) and with NDMA treatment (**C** and **D**). The images are representative of eight mice per group. Note the marked contrast of α‐SMA‐positive stellate cells in NDMA‐treated WT (**C**) and MMP‐13 KO mouse livers (**D**). (**E**) Quantitative evaluation of activated stellate cells. Staining intensity of α‐SMA‐positive cells was analysed using Image‐pro discovery software. The data are mean ± S.D. of 10 randomly selected microscopic fields from eight mice per group. ***P < 0.001 NDMA‐treated WT mice *versus* control untreated WT mice; **P < 0.01 NDMA‐treated MMP‐13 KO mice *versus* untreated MMP‐13 KO mice; ^#^ *P* < 0.001 NDMA‐treated MMP‐13 KO mice *versus* NDMA‐treated WT mice. Original magnification, ×100.

Immunohistochemical staining was performed for α‐SMA to examine the activation of hepatic stellate cells after induction of liver injury by NDMA. As shown in Figure 3A and B, the staining was almost completely absent in control WT and MMP‐13 KO mice without the treatment. However, marked staining of α‐SMA appeared in stellate cells localized in and around the necrotic and fibrotic areas of the liver from NDMA‐treated WT mice (Fig. 3C), although minimal staining was observed in NDMA‐treated MMP‐13 KO mice (Fig. 3D). Quantitative analysis of the staining intensity of α‐SMA demonstrated that the intensity is significantly higher in NDMA‐treated WT mice as compared to control non‐treated WT mice (*P* < 0.001) (Fig. 3E). It was also significantly higher in NDMA‐treated MMP‐13 KO mice than in non‐treated MMP‐13 KO mice (*P* < 0.01) (Fig. 3E). However, when the staining intensity was compared between the WT and MMP‐13 KO mouse groups, it was significantly lower in the MMP‐13 KO mice than in the WT mice (*P* < 0.001) (Fig. 3E).

### Expression of CTGF after NDMA treatment is reduced in MMP‐13 KO mice

**Figure 4:**
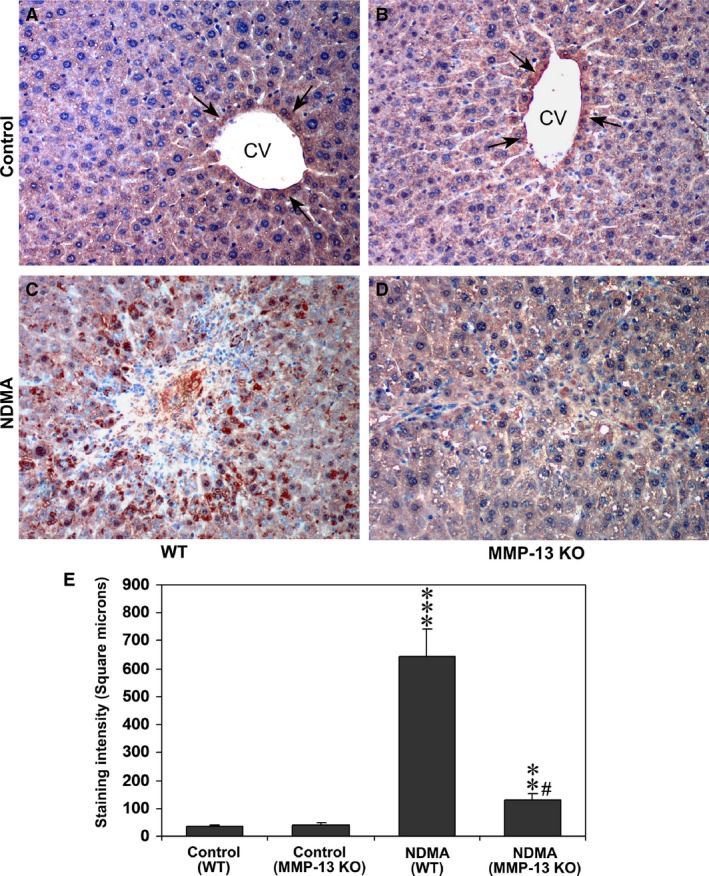
Immunohistochemical staining of CTGF in the liver tissues from WT and MMP‐13 KO mice without and with NDMA treatment. The images are representative of eight mice per group. (**A** and **B**) WT and MMP‐13 KO mice without NDMA treatment, respectively. Arrows, weak staining of CTGF in one row of the peri‐central hepatocytes. (**C** and **D**) NDMA‐treated WT and MMP13 KO mice, respectively. Note that marked contrast in number of the CTGF‐stained cells between WT (**C**) and MMP‐13 KO mice (**D**). (**E**) Quantification of CTGF staining using Image‐pro discovery software. The data are mean ± S.D. of 10 randomly selected microscopic fields from eight mice per group. ***P < 0.001 NDMA‐treated WT mice *versus* control untreated WT mice; **P < 0.01 NDMA‐treated MMP‐13 KO mice *versus* untreated MMP‐13 KO mice; ^#^ *P* < 0.001 NDMA‐treated MMP‐13 KO mice *versus* NDMA‐treated WT mice. Original magnification, ×100.

The expression of CTGF in the livers from control and NDMA‐treated WT and MMP‐13 KO mice was determined by immunohistochemistry. The staining was absent or negligible in the parenchymal areas of control WT and MMP‐13 KO mice, but weak staining was obtained in one row of the peri‐central hepatocytes in both WT and MMP‐13 KO mice (arrows) (Fig. 4A and B), the finding coinciding with the previous report 22. On the other hand, marked and intense staining was present by the parenchymal cells in the necrotic and fibrotic areas in NDMA‐treated WT mice (Fig. 4C), although only focal minimal staining was observed in the livers from NDMA‐treated MMP‐13 KO mice (Fig. 4D). The staining intensity of CTGF was significantly higher in NDMA‐treated WT mice compared to the untreated WT control mice (*P* < 0.001) (Fig. 4E). Although the staining intensity was significantly higher in NDMA‐treated MMP‐13 KO mice compared to the MMP‐13 KO control untreated mice (*P* < 0.01), the intensity in NDMA‐treated MMP‐13 KO mice was significantly lower than that in the NDMA‐treated WT mice (*P* < 0.001) (Fig. 4E).

### Interleukin‐6 levels in mouse serum

**Figure 5:**
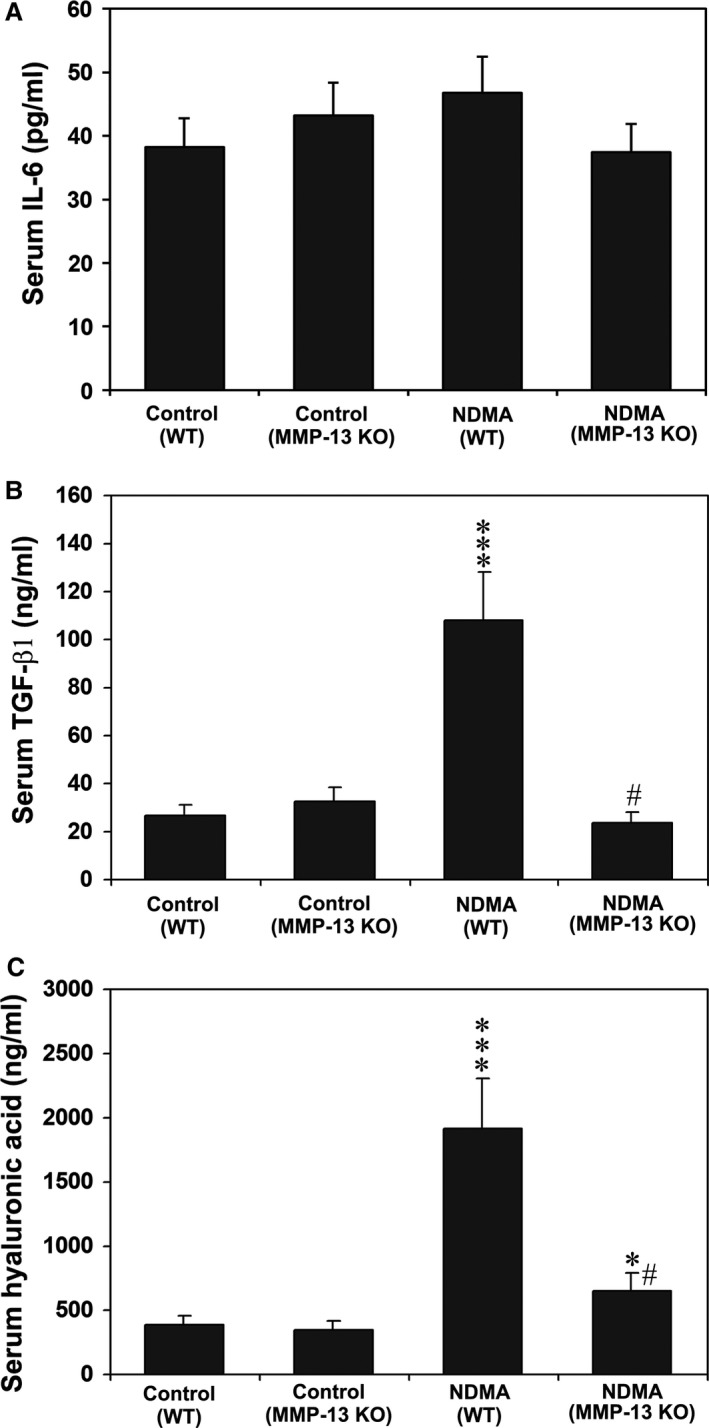
Serum levels of IL‐6, TGF‐β1 and HA in WT and MMP‐13 KO mice treated without and with NDMA. The data are mean ± S.D. of eight mice per group. ***P < 0.001 NDMA‐treated WT mice *versus* control untreated WT mice; *P < 0.05 NDMA‐treated MMP‐13 KO mice *versus* untreated MMP‐13 KO mice; ^#^ *P* < 0.001 NDMA‐treated MMP‐13 KO mice *versus* NDMA‐treated WT mice.

The acute phase protein, IL‐6, was measured in the mouse serum to exclude chronic inflammation during the i.p. procedure. There was no significant difference in IL‐6 levels between the groups of animals involved in the study (Fig. 5A). The data ruled out peritonitis or any systemic infection during the course of the study that could affect the outcome of the results.

### Serum levels of TGF‐β1 and HA decreased in MMP‐13 KO mice

TGF‐β1 is known to be up‐regulated during liver injury and fibrosis 21, and serum HA is a very early indicator of liver damage 6, 37. Thus, we measured serum levels of TGF‐β1 and HA in WT and MMP‐13 KO mice. As shown in Figure 5B and C, both TGF‐β1 and HA levels significantly increased in NDMA‐treated WT mice as compared to control untreated WT and MMP‐13 KO mice. However, the TGF‐β1 and HA levels were decreased 4‐fold in NDMA‐treated MMP‐13 KO mice than in NDMA‐treated WT mice (*P* < 0.001) (Fig. 5B and C).

### Activity and expression of MMP‐9 reduced in MMP‐13 KO mice

**Figure 6:**
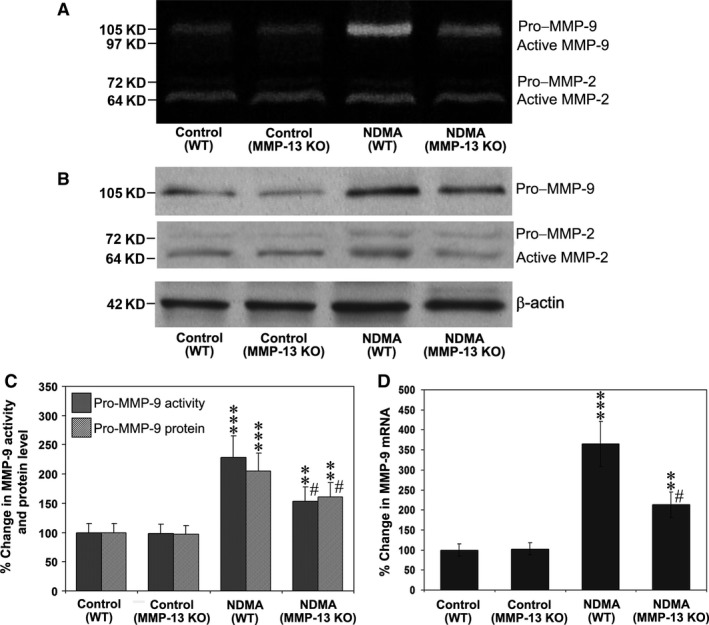
Expression of MMP‐9 and MMP‐2 in WT and MMP‐13 KO mice treated without and with NDMA. (**A**) Gelatin zymography for MMP‐9 and MMP‐2 in supernatants of the liver tissues from WT and MMP‐13 KO mice. Pro‐MMP‐9 of 105 kD, active MMP‐9 of 97 kD, pro‐MMP‐2 of 72 kD and active MMP‐2 of 64 kD are indicated. (**B**) Western blotting for proteins of MMP‐9 and MMP‐2 in the liver tissue. β‐Actin was used as a loading control. (**C**) Quantitative densitometric analyses of MMP‐9 activity and protein level. The data are mean ± S.D. of eight mice per group. ***P < 0.001 NDMA‐treated WT mice *versus* control untreated WT mice; **P < 0.01 NDMA‐treated MMP‐13 KO mice *versus* untreated MMP‐13 KO mice; ^#^ *P* < 0.001 NDMA‐treated MMP‐13 KO mice *versus* NDMA‐treated WT mice. (**D**) Real‐time PCR analysis for the quantitative expression of MMP‐9 mRNA in WT and MMP‐13 KO. The data are mean ± S.D. of eight mice per group. ***P < 0.001 NDMA‐treated WT mice *versus* control untreated WT mice; **P < 0.01 NDMA‐treated MMP‐13 KO mice *versus* untreated MMP‐13 KO mice; ^#^ *P* < 0.001 NDMA‐treated MMP‐13 KO mice *versus* NDMA‐treated WT mice.

The activity of MMP‐9 and MMP‐2 were measured by gelatin zymography in the liver samples from WT and MMP‐13 KO mice. As shown in Figure 6A and C, MMP‐9 activity was significantly increased in NDMA‐treated WT mice as compared to control WT mice (*P* < 0.001) or in NDMA‐treated MMP‐13 KO mice compared to untreated MMP‐KO mice (*P* < 0.01). However, MMP‐9 activity in NDMA‐treated MMP‐13 KO mice was significantly lower (*P* < 0.001) compared to the NDMA‐treated WT mice (Fig. 6C). Figure 6B depicts the Western blots for MMP‐9 and MMP‐2 proteins in control WT and MMP‐13 KO mice and NDMA‐treated mice, showing a similar pattern obtained by the gelatin zymography. MMP‐9 protein level appeared to be less in NDMA‐treated MMP‐13 KO mice as compared to NDMA‐treated WT mice (Fig. 6B), and quantitative analysis confirmed the data as shown in Figure 6C. The expression of MMP‐9 mRNA in control WT and MMP‐13 KO mice and the NDMA‐treated mice is shown in Figure 6D. Expression of MMP‐9 mRNA was significantly increased in NDMA‐treated WT (*P* < 0.001) and MMP‐13 KO (*P* < 0.01) mice compared to their respective untreated controls. However, the mean MMP‐9 mRNA level in NDMA‐treated MMP‐13 KO mice was significantly lower (*P* < 0.001) compared to NDMA‐treated WT mice.

### Deletion of MMP‐13 decreases expression of growth factors and proteins that trigger hepatic fibrosis

**Figure 7:**
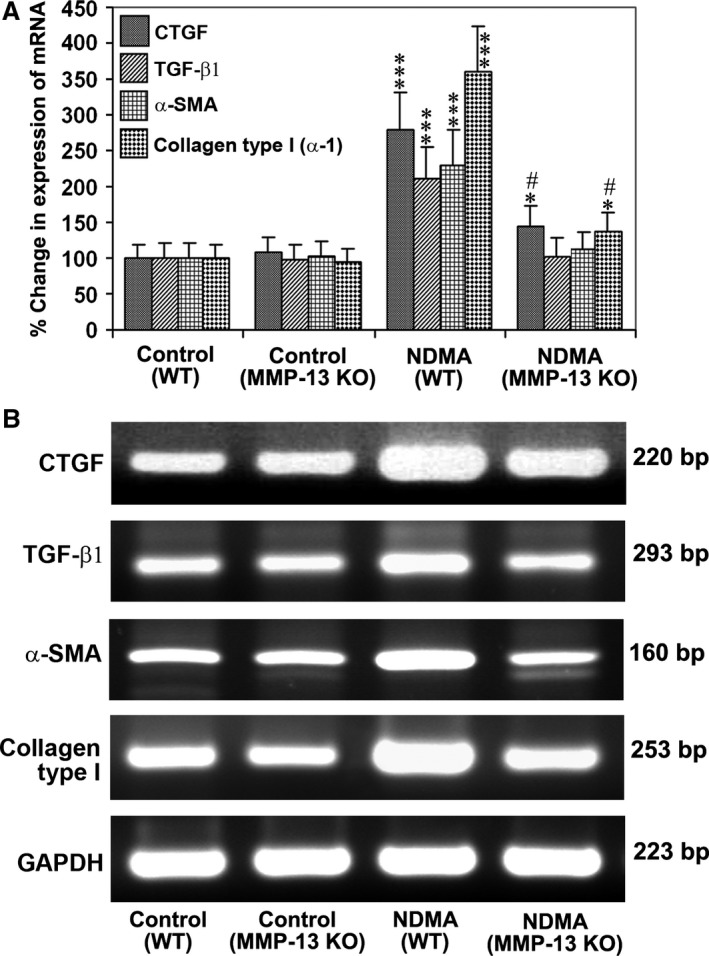
Expression of CTGF and TGF‐β1 in WT and MMP‐13 KO mice treated without and with NDMA. (**A**) Real‐time PCR analysis for the quantitative expression of CTGF, TGF‐β1, α‐SMA and collagen type 1 mRNA in WT and MMP‐13 KO mice. The data are mean ± S.D. of six samples per group. ***P < 0.001 NDMA‐treated WT mice *versus* control untreated WT mice; *P < 0.05 NDMA‐treated MMP‐13 KO mice *versus* untreated MMP‐13 KO mice; ^#^ *P* < 0.001 NDMA‐treated MMP‐13 KO mice *versus* NDMA‐treated WT mice. (**B**) Semiquantitative RT‐PCR for the expression of CTGF, TGF‐β1, α‐SMA and type 1 collagen mRNA in WT and MMP‐13 KO mice treated without and with NDMA. GAPDH was used as a loading control. The data are representative of six samples in each group.

To examine the expression of CTGF, TGF‐β1, α‐SMA and type I collagen, the respective mRNA levels were quantified using real‐time PCR in control WT and MMP‐13 KO mice and NDMA‐treated mice. All of the molecules studied were significantly elevated (*P* < 0.001) in NDMA‐administered WT mice (Fig. 7A). The levels of CTGF and type I collagen were also significantly increased (*P* < 0.05) in NDMA‐treated MMP‐13 KO mice as compared to control MMP‐13 KO mice, but there was no significant increase in the levels of TGF‐β1 or α‐SMA (Fig. 7A). The increased mRNA levels of CTGF and type I collagen in NDMA‐treated MMP‐13 KO mice were significantly (*P* < 0.001) lower as compared to the NDMA‐treated WT mice. Semiquantitative RT‐PCR analysis of CTGF, TGF‐β1, α‐SMA and type I collagen mRNA in the liver specimens from WT and MMP‐13 KO confirmed the data of real‐time PCR (Fig. 7B).

### Deletion of MMP‐13 decreases protein levels of major molecules involved in hepatic fibrosis

**Figure 8:**
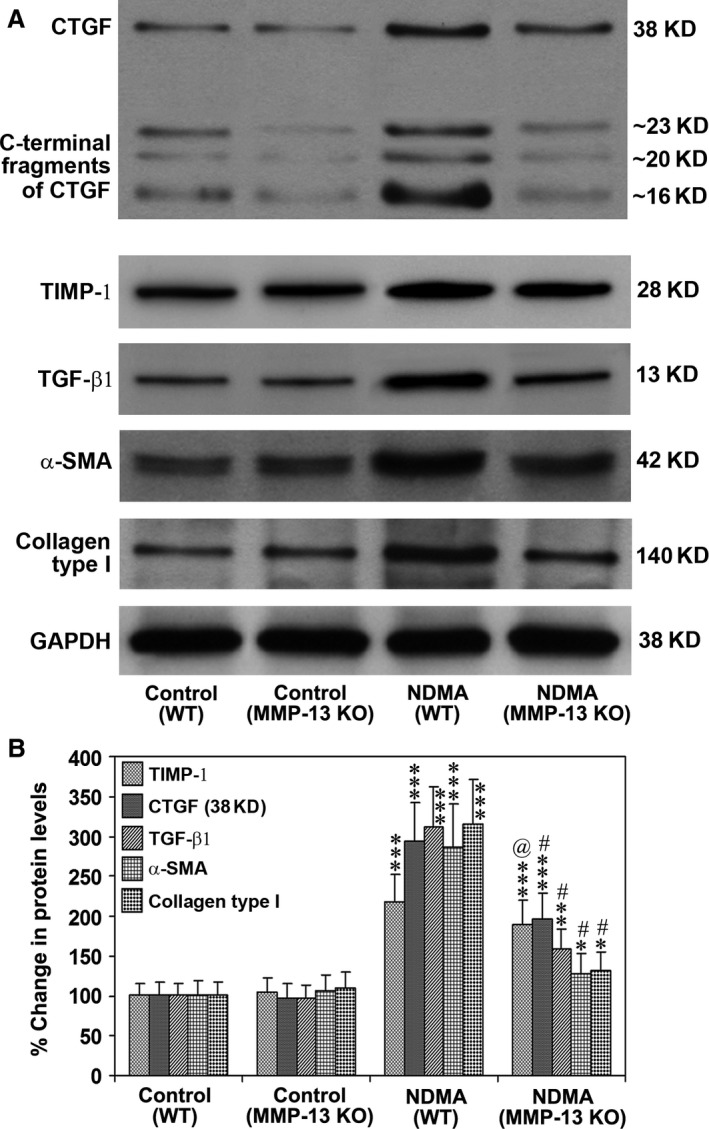
Protein expression of CTGF, TIMP‐1, TGF‐β1, α‐SMA and collagen type 1 in WT and MMP‐13 KO mice treated without and with NDMA. (**A**) Western blotting for the proteins of full‐length CTGF and its C‐terminal fragments, TIMP‐1, active TGF‐β1, α‐SMA and collagen type 1 in the liver tissues from WT and MMP‐13 KO mice. The images are representative of six Western blots per each group. (**B**) Quantitative analysis of Western blot images. The data are mean ± S.D. of six images per group. ***P < 0.001 NDMA‐treated WT mice *versus* control untreated WT mice and NDMA‐treated MMP‐13 KO mice *versus* untreated MMP‐13 KO mice; **P < 0.01 NDMA‐treated MMP‐13 KO mice *versus* untreated MMP‐13 KO mice; *P < 0.05 NDMA‐treated MMP‐13 KO mice *versus* untreated MMP‐13 KO mice; ^#^ *P* < 0.001 NDMA‐treated MMP‐13 KO mice *versus* NDMA‐treated WT mice; @, *P* < 0.05 NDMA‐treated MMP‐13 KO mice *versus* NDMA‐treated WT mice.

The protein levels of full‐length CTGF and its cleaved products as well as TIMP‐1, cleaved TGF‐β1, α‐SMA and type I collagen were determined by Western blotting. There was an increase in full‐length CTGF in both NDMA‐treated WT and MMP‐13 KO mice. The increase appeared to be higher in WT mice compared to MMP‐13 KO mice (Fig. 8A). The cleaved fractions of CTGF were increased only in NDMA‐treated WT mice but not in MMP‐13 KO mice, suggesting a role of MMP‐13 in the cleavage of CTGF. Figure 8B represents the quantification of Western blot images, which is presented as percentage alteration of the proteins. There was a marked increase (*P* < 0.001) in protein levels of CTGF, TIMP‐1, cleaved TGF‐β1, α‐SMA and type I collagen in NDMA‐treated WT mice as compared to those in control untreated WT mice. Protein levels of TIMP‐1 (*P* < 0.001), full‐length CTGF (*P* < 0.001), TGF‐β1 (*P* < 0.01), α‐SMA (*P* < 0.05) and type I collagen (*P* < 0.05) were significantly increased in NDMA‐treated MMP‐13 KO mice as compared to control untreated MMP‐13 KO mice. However, the mean protein levels of all these molecules were significantly lower (*P* < 0.001) (TIMP‐1, *P* < 0.05) in NDMA‐treated MMP‐13 KO mice compared to NDMA‐treated WT mice (Fig. 8B). Among all the five molecules studied, the maximum elevation (3.12‐fold) was observed in the case of type I collagen in NDMA‐treated WT mice (Fig. 8B). Reprobing the blots for GAPDH demonstrated loading of similar levels of proteins in all lanes.

### Inhibition of MMP‐13 in hepatic stellate cells decreases CTGF and TGF‐β1 protein levels

**Figure 9:**
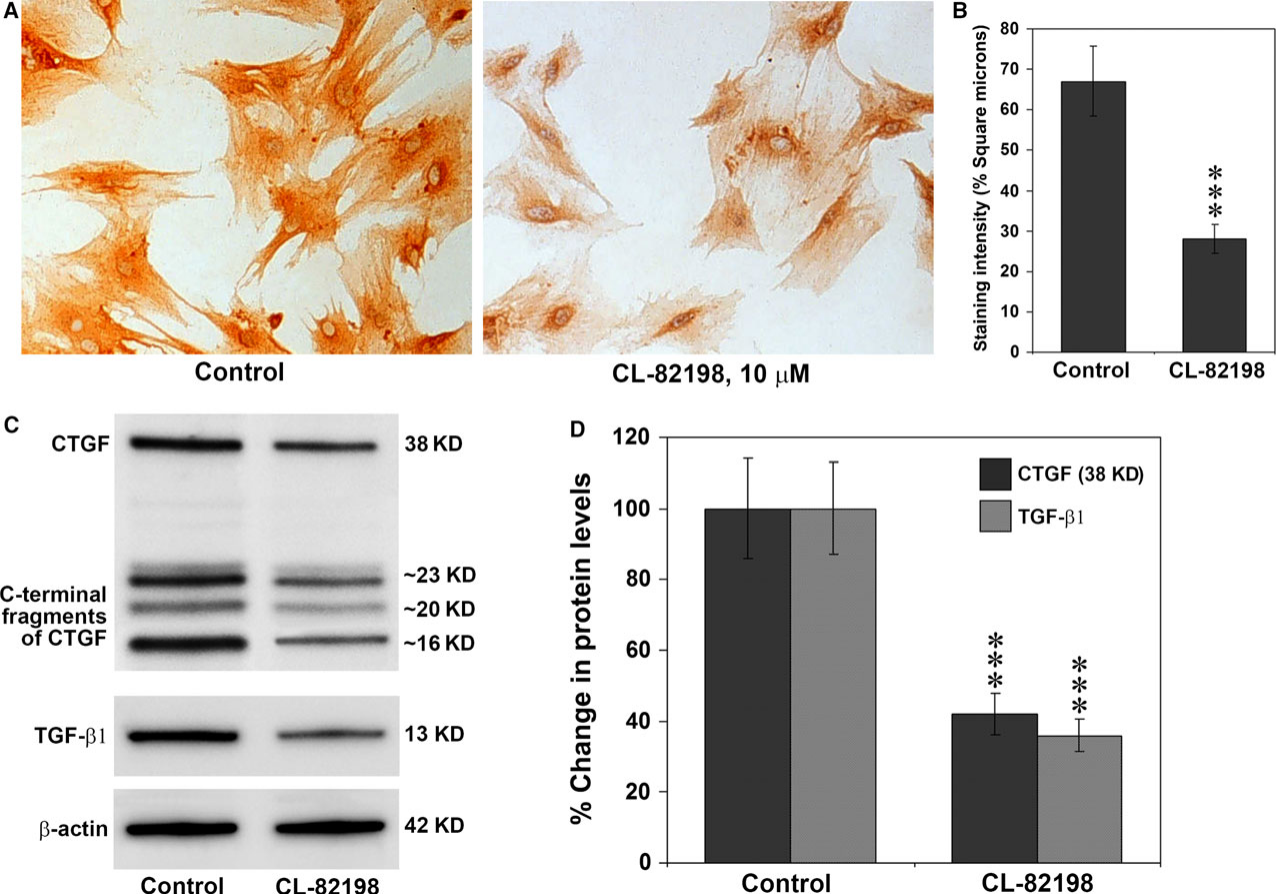
Protein expression of CTGF and TGF‐β1 in cultured rat hepatic stellate cells. (**A**) Immunohistochemical staining for CTGF in cultured rat hepatic stellate cells. Marked and strong staining for CTGF was present in the activated hepatic stellate cells. Treatment with 10 μM CL‐82198 (final concentration) in the culture media resulted in a marked decrease of CTGF staining intensity. Original magnification, ×200. (**B**) Quantification of the staining intensity of CTGF in cultured stellate cells using Image‐pro discovery software. The data are mean ± S.D. of 10 randomly selected microscopic fields from five culture slides. ***P < 0.001 CL‐82198 treated cultures *versus* untreated cultures. (**C**) Western blotting for the proteins of full‐length CTGF and its C‐terminal fragments and active TGF‐β1 in hepatic stellate cells untreated and treated with CL‐82198. The images are representative of five Westerns per group. (**D**) Quantitative analysis of CTGF and TGF‐β1 Western blot images. The data are mean ± S.D. of five Western blot images per group. ***P < 0.001 CL‐82198‐treated cultures *versus* untreated cultures.

To obtain more information about the mechanism of decreased liver injury and fibrosis observed in MMP‐13 knockout mice, we isolated hepatic stellate cells from rat liver and cultured. The activated stellate cells were treated with CL‐82198 to inhibit MMP‐13 activity. Immunohistochemical staining for CTGF demonstrated conspicuous staining for CTGF in untreated stellate cells and a marked decrease of staining intensity in the cells treated with CL‐82198 (Fig. 9A). Quantitative analysis of CTGF staining intensity depicted around 60% decrease in the treated cells compared to the untreated cells (Fig. 9B). Western blot analysis depicted that blocking of MMP‐13 activity resulted in a marked decrease of full‐length CTGF and as well as its cleaved fragments and also the active subunit of TGF‐β1 indicating MMP‐13 is required for the cleavage of both CTGF and TGF‐β1 (Fig. 9C). Figure 9D represents the quantification of the Western blot images of full‐length CTGF and active TGF‐β1, which is presented as percentage alteration of the proteins. Compared to the untreated cells, there was a significant decrease (*P* < 0.001) in both CTGF and TGF‐β1 protein levels in CL‐82198 treated cells. Reprobing the Western blots for β‐actin demonstrated equal loading of proteins in both lanes.

## Discussion

In the present study, we have demonstrated that loss of MMP‐13 attenuates NDMA‐induced liver injury and fibrosis in a mouse model, and suggested that MMP‐13 is involved in the accelerated inflammation by overexpression and activation of CTGF in the impaired liver. Although the beneficial effects of collagenolytic MMPs such as MMP‐1 and MMP‐8 on liver fibrosis have been documented 38, 39 previous study on a model of the biliary duct ligation‐induced cholestasis and fibrosis using MMP‐13 KO mice showed a promotional effect of MMP‐13 on the initial inflammatory response and subsequent fibrosis 29. This study provided the data that MMP‐13 has a role in the expression of pro‐inflammatory mediators such as tumour necrosis factor‐α (TNF‐α) and Chemokine (C‐C motif) ligand 2 (CCL2), which modulate the initial liver damage in their model. The present study not only confirmed the data of promoting effect of MMP‐13 on the initial liver damage and fibrosis but also provided the first evidence that CTGF overexpression and activation by MMP‐13 are implicated in the process.

MMP‐13 is a zinc‐containing interstitial collagenase capable of degrading native fibrillar collagens and plays a key role in the remodelling of connective tissue matrix under both physiological and pathological conditions 16, 40. In normal and fibrotic livers, expression of collagen types I, III and IV takes place predominantly in non‐parenchymal cells 41. During early fibrogenesis, the hepatic fibrillar collagens are first cleaved by MMP‐13 29 and the partially denatured collagens are subsequently digested by gelatinolytic activities of other MMPs such as MMP‐9 42. We observed that the mRNA and protein expression of MMP‐9 and its activity are markedly increased in NDMA‐treated WT mice, and the levels are significantly suppressed in NDMA‐treated MMP‐13 KO mice. In addition, α‐SMA‐positive stellate cells were substantially reduced in the liver tissue from NDMA‐treated MMP‐13 KO mice. Thus, these data strongly suggest that MMP‐13 and MMP‐9 are involved in the degradation of hepatic ECM in the initial phase of hepatic fibrosis and pave the way for the proliferation of activated hepatic stellate cells that transform into myofibroblasts.

The deposition of cross‐linked fibrillar collagens, especially collagens types I and III, in the extracellular space of the liver accompanied by defenestration of endothelial cells is the hallmark of hepatic fibrosis 2, 8, 43. Previous studies have suggested the implications of CTGF in the pathogenesis of hepatic fibrosis characterized by deposition of fibrillar collagens in the liver 21, 44, 45, 46. The full‐length CTGF molecule comprises four structural domains, that is modules 1–4, which are susceptible to proteolysis by hepatic stellate cells, yielding C‐terminal fragments composed of the modules 3 and 4 or the module 4 alone 46. Peptide mapping and site‐directed mutagenesis study indicated that the sequence I^257^RTPKISKPIKFELSG^272^ in the C‐terminal region of CTGF is a unique binding domain for integrin αvβ3 that is sufficient to mediate integrin αvβ3‐ and heparan sulphate proteoglycan‐dependent (HSPG) stellate cell adhesion 46. The N‐terminal CTGF fragments are reported to be a marker of the fibrotic phenotype in fibrotic diseases such as scleroderma, and both N‐terminal and C‐terminal fragments of CTGF are suggested to be more potent fibrogenic stimulators compared to the full‐length parent molecule 28. In the present study, we have demonstrated the generation of the C‐terminal fragments of CTGF, besides full‐length CTGF, in the liver tissues from NDMA‐treated WT mice by immunoblotting using the antibody specific to the I^257^RTPKISKPIKFELSG^272^ peptide, and their expression was reduced in NDMA‐treated MMP‐13 KO mice. Importantly, the inflammation, necrosis, activation of stellate cells, and deposition of collagen fibres were also substantially suppressed in NDMA‐treated MMP‐13 KO mice. These data suggest that MMP‐13 plays a central role in the early inflammatory response through modulation of CTGF expression and processing during the pathogenesis of hepatic fibrosis.

One of the interesting findings in our study is that overproduction of CTGF within the liver tissues of NDMA‐treated WT mice was associated with the overexpression of active TGF‐β1. TGF‐β1 is produced by cells as an inactivated complex form containing latency‐associated peptide (LAP) and latent TGF‐β‐binding protein (LTBP), and activated to the cleaved active TGF‐β1 by several pathways, which include the processing by MMP species such as MMP‐2 and MMP‐9 47. In the present study, we could show the activated form of TGF‐β1 of 13 kD by immunoblotting of the liver tissues, and a previous study on skin wound healing in MMP‐13 KO mice demonstrated that MMP‐13 plays a key role in the activation of latent TGF‐β1 31. Thus, it may be possible to speculate the implication of MMP‐13 in the activation of TGF‐β1 within the liver tissues, although the exact activation mechanism of latent TGF‐β1 in the present study remains to be clarified. Both CTGF and TGF‐β1 exhibit a profound interaction during the pathogenesis of hepatic fibrosis and concurrently elevate in animal models of fibrosis 23, 48. CTGF was originally reported to be transcriptionally induced by TGF‐β1 49. In human skin fibroblasts, high‐level expression of CTGF mRNA and protein is observed only by stimulation with TGF‐β1 among several growth factors including TGF‐β1, platelet‐derived growth factor, epidermal growth factor and basic fibroblast growth factor 50. Nevertheless, the related expression patterns of CTGF and TGF‐β1 suggest the possibility that these two molecules act synergistically for the NDMA‐induced hepatic injury and fibrosis.

*In vitro* studies using isolated hepatic stellate cells from rat liver proved that MMP‐13 is involved in the cleavage of both CTGF and TGF‐β1, thus play a significant role in the pathogenesis of hepatic fibrosis through modulation of the processing of profibrogenic molecules. CL‐82198 is a very specific inhibitor of MMP‐13 without affecting the activity of MMP‐1 or MMP‐9 and blocks 89% of MMP‐13 activity at a concentration of 10 μg/ml 35. As in *in vivo* studies, inhibition of MMP‐13 resulted in a significant decrease of C‐terminal fragments of CTGF using the specific antibody that detects C‐terminal cleaved subunits. It is important to note that blocking of MMP‐13 resulted in a decrease of around 60% of active TGF‐β1, which could answer the significant decrease of 38 kD full‐length CTGF in the treated cells. It is well established that TGF‐β1 regulates the expression of CTGF and TGF‐β1 is a requirement for the induction of CTGF 51. The results of the current *in vitro* studies further demonstrate that both TGF‐β1 and CTGF acts synergistically as in *in vivo* system and could be the most probable reason for the decrease of 38 kD full‐length CTGF protein in CL‐82198 treated hepatic stellate cells.

In summary, we have demonstrated that the NDMA‐induced inflammation and injury and subsequent fibrosis of the liver are substantially decreased in MMP‐13 KO mice, and these effects of MMP‐13 involve the expression and processing of CTGF and TGF‐β1. Our study suggests that MMP‐13 promotes hepatic damage and fibrosis by modulation of these growth factors within the liver tissues. Thus, blocking of the expression and/or activation of CTGF in early stages of liver injury may pave a way to prevent the progression of hepatic damage and fibrosis.

## Author contribution

Joseph George carried out all the major experiments, collected the data, analysed and interpreted the data, and wrote the manuscript. Mikihiro Tsutsumi obtained funding, provided technical and material support, and participated in drafting the manuscript. Mutsumi Tsuchishima involved in research design, analysis and interpretation of the data, and critical review.

## Conflict of interests

The authors do not have any conflict of interests to declare in connection with this manuscript.
